# Solvent-sensitive nanoparticle-enhanced PCR assay for the detection of enterotoxigenic *Escherichia coli*

**DOI:** 10.1038/s41598-022-25088-3

**Published:** 2022-11-30

**Authors:** Patcharapong Teawprasong, Yodsathorn Wongngam, Tienrat Tangchaikeeree, Abdelhamid Elaissari, Pramuan Tangboriboonrat, Duangporn Polpanich, Kulachart Jangpatarapongsa

**Affiliations:** 1 Center for Research and Innovation, Faculty of Medical Technology, Mahidol University, Nakhon Pathom, 73170 Thailand; 2 National Nanotechnology Center, National Science and Technology Development Agency (NSTDA), Thailand Science Park, Pathum Thani, 12120 Thailand; 3 Univ Lyon, University Claude Bernard Lyon-1, CNRS, ISA-UMR 5280, 69622 Villeurbanne, France; 4 Department of Chemistry, Faculty of Science, Mahidol University, Rama 6 Road, Phyathai, Bangkok, 10400 Thailand

**Keywords:** Biotechnology, Microbiology, Nanoscience and technology

## Abstract

Stimulus-responsive nanoparticles are among the most utilized nanoscale materials in biomedical applications. As these nanoparticles exhibit a manipulable response to a particular stimulus, such as pH, heat, and organic solvent, they are potential signalling units in diagnostic assays. This study aims to enhance the limit of detection and reduce the turnaround time of magnetic nanoparticle polymerase chain reaction (PCR) enzyme-linked gene assay (MELGA), an advanced PCR-based technique termed the solvent-sensitive nanoparticle (SSNP)-enhanced PCR assay. This technique was proposed to detect pathogenic enterotoxigenic *Escherichia coli* (ETEC) through applying stimulus-responsive nanoparticles. The SSNPs were elaborated with three main components, including mesoporous silica nanoparticles as a structural unit, organic dye (Nile red) as a payload, and the corresponding organic solvent-sensitive polymer shell as “gatekeeper” (poly(maleic anhydride-*alt*-methyl vinyl ether, PMAMVE). A suitable organic solvent capable of inducing polymer swelling and dye dissolution was investigated by considering a solubility parameter. Using ethanol, the encapsulated Nile red can diffuse out of the SSNPs faster than other solvents and reach a constant concentration within 15 min. For the PCR inhibition study, various SSNPs concentrations (10–30 μg/reaction) were mixed with the ETEC gene and PCR reagent. The results showed that the particles in this concentration range did not inhibit PCR. By comparing the efficacy of conventional PCR, MELGA, and SSNP-enhanced PCR assay, the proposed technique showed a better detection limit than that of PCR, whereas that of MELGA was the lowest. Moreover, compared to MELGA or conventional PCR, this technique provided remarkably faster results in the postamplification process.

## Introduction

Molecular assays for DNA amplification, e.g., polymerase chain reaction (PCR), are highly efficient in terms of their sensitivity and specificity in detecting targeted DNA^1^. Several studies have reported the development of PCR-based alternatives. For example, to increase the sensitivity of detection, magnetic nanoparticles (MNPs) with superparamagnetic properties and miscellaneous functionalization have been applied to isolate nucleic acids before amplification^2–5^. Notably, the newly developed PCR-based technique, which was termed magnetic nanoparticles PCR enzyme-linked gene assay (MELGA), was introduced in the recent decade^6–9^. By applying forwards primer-modified MNPs to enrich the PCR product and biotinylated reverse primers to dramatically improve the sensitivity of postamplification analysis, MELGA could provide a limit of detection at the femtogram level^7^. According to a previous study, this technique exhibited 1,000 times higher sensitivity than that of conventional PCR^7,8^. Nevertheless, despite the high sensitivity, the signal developing step in MELGA postamplification was time-consuming due to multiple washing steps and prolonged reaction times between biotin-streptavidin and the enzyme–substrate. Thus, MELGA must be further improved, especially in terms of the processing time to quicken the turnaround time and simplify the postamplification process.

In recent years, stimulus-responsive nanoparticles have gained much attention as nanoscale drug delivery systems, especially in the biomedical field^10^. These nanoparticles can alter their “gatekeeper” polymeric structure to function as a valve, resulting in a controllable payload release that is dependent on stimuli. For instance, pH-responsive nanoparticles remain intact in most conditions and their system is altered in a particular situation or at a specific pH, producing a response that releases the cargo. This unique characteristic enhances the efficacy for therapeutic purposes^10,11^.

**Figure 1:**
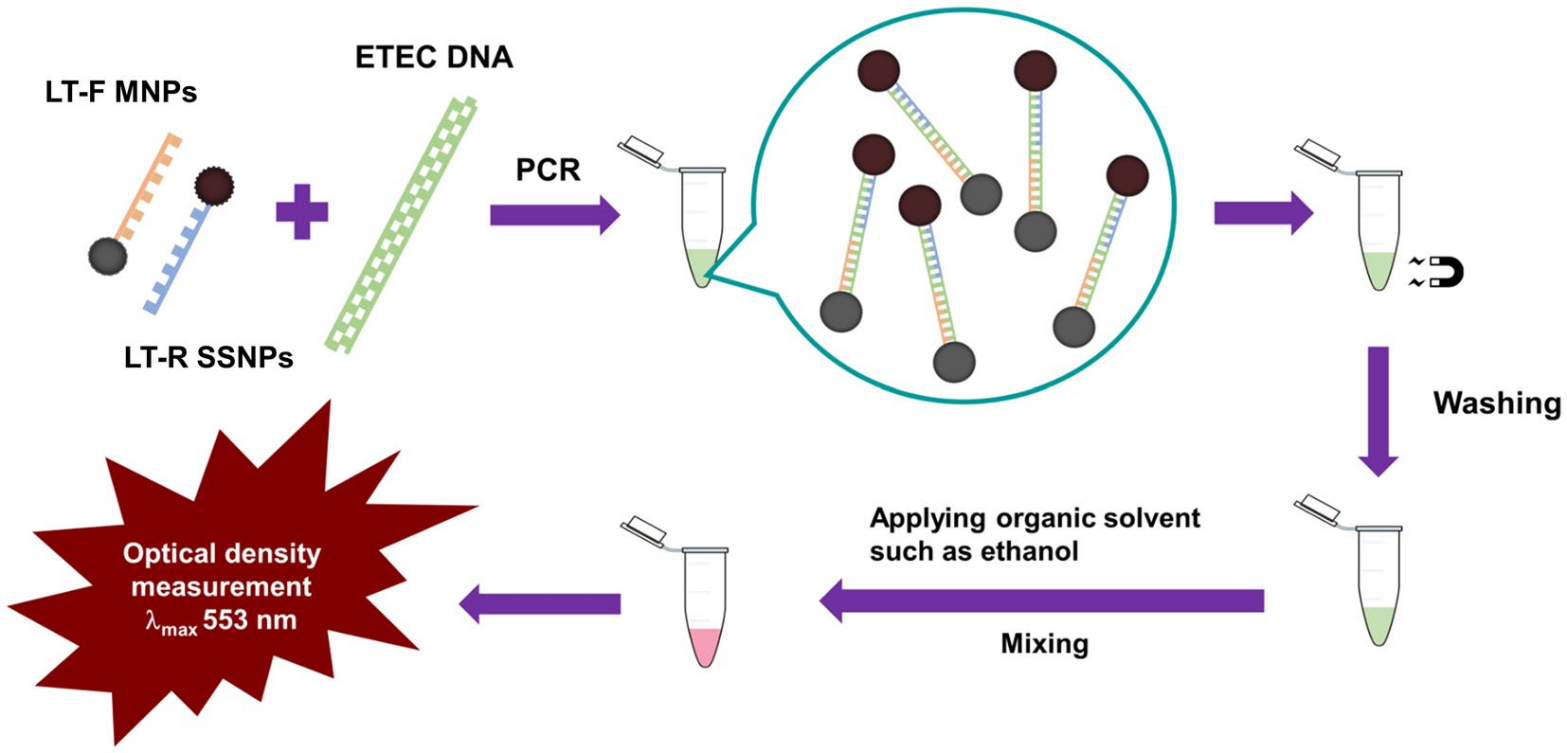
Schematic representation of the solvent-sensitive nanoparticle (SSNP)-enhanced PCR assay for the detection of enterotoxigenic *Escherichia coli*.

In this study, solvent-sensitive nanoparticles (SSNPs), a kind of stimulus-responsive nanoparticle, were first developed to enhance the efficiency of MELGA and detect pathogenic enterotoxigenic *Escherichia coli* (ETEC). This pathogen causes more than 400 million diarrhoeagenic cases per year, resulting in 300,000–500,000 casualties, which mostly occur in young children in developing countries^12–16^. Since PCR-based molecular assays are the only way to distinguish ETEC from other *E. coli*^12,16,17^, these assays need to be improved for efficient detection^13,18^. Here, a technique named the solvent-sensitive nanoparticle (SSNP)-enhanced PCR assay was proposed. The SSNPs were synthesized by loading Nile red dye into mesoporous silica nanoparticles (MSNs) and coated with poly(maleic anhydride-*alt*-methyl vinyl ether) (PMAMVE) copolymer as a gatekeeper. A forwards primer corresponding to the *LT* gene (LT-F), a heat-labile toxin gene of ETEC, was immobilized onto the surface of carboxylated MNPs (LT-F-MNPs) using the carbodiimide method for a separating unit. A reverse primer (LT-R) that corresponded to the same targeted gene was covalently bound to the surface of SSNPs (LT-R-SSNPs) for the signalling unit. PCR was performed by mixing the DNA extracted from a certain concentration of ETEC using the DNA extraction standard technique, LT-F MNPs and LT-R SSNPs under recommended conditions with a thermocycler. Amplicons were separated and washed twice using external magnetic fields. Finally, an optimal organic solvent, such as ethanol, which encouraged the swelling of PMAMVE and then dye dissolution, was added and mixed well for 10–15 min. The optical density of the dye in the supernatant related to the amount of amplicons was measured at a wavelength of 553 nm. The principle of the proposed technique is depicted in Fig. 1. It was found that SSNPs are compatible with PCR and can quickly provide colorimetric results. By utilizing SSNPs, the incubation period for signal-developing interactions in the postamplification process was significantly reduced compared to that of conventional PCR and MELGA. Therefore, the newly developed PCR assay enhanced by SSNP is a promising, rapid, and efficient method for diagnostic purposes, especially for investigating pathogenic microorganisms.

## Materials and methods

### Instruments and reagents

Mesoporous silica nanoparticles (NH_2_-MSNs) and 9-(diethylamino)-*5H*-benzo[*a*]phenoxazin-5-one (Nile red) were purchased from Sigma‒Aldrich, USA. PMAMVE was purchased from Polysciences Inc., USA. Carboxylated magnetic nanoparticles (COOH-MNPs) (1.45 wt%, hydrodynamic size 250 nm, zeta potential − 40 mV) were kindly provided by Claude Bernard University Lyon 1, France. Bacterial isolates of ETEC (DMSC 25093) were acquired from the Department of Medical Science, Thailand. The Presto™ Mini gDNA Bacteria Kit for bacterial DNA extraction was purchased from Geneaid, Taiwan. A pair of primers of heat-labile enterotoxin encoding the *LT* gene for conventional PCR, MELGA and SSNPs-enhanced PCR assay, i.e., the specific LT forwards primer (5′-GGC GAC AGA TTA TAC CGT-3′ and 5′-NH_2_-C_6_-GGC GAC AGA TTA TAC CGT-3′) and LT reverse primer (5′-CGG TCT CTA TAT TCC CTG-3′, biotin-5′-CGG TCT CTA TAT TCC CTG-3′ and 5′-NH_2_-C_6_-CGG TCT CTA TAT TCC CTG-3′), were designed using Primer-BLAST software and synthesized from Bio Basic Canada Inc., Canada^8,19^. 2-(*N*-Morpholino)ethane sulfonic acid (MES) and *N*-(3-dimethylaminopropyl)-*N*′-ethylcarbodiimide (EDC) were purchased from Sigma‒Aldrich, USA, and used for primer immobilization. 1 × Accustart II PCR Supermix and staining agent (GelRed) were purchased from Quantabio and Biotium, USA. Horseradish peroxidase conjugated streptavidin (SA-HRP) (Kierkegaard & Perry Laboratories Inc, USA) and TMB substrate (3, 3′, 5, 5′-tetramethylbenzidine) (Thermo Fisher Scientific Inc., USA) were used for MELGA. Deionized (DI) water was used throughout the study. The average hydrodynamic size and zeta potential of the synthesized SSNPs were confirmed by a Nanosizer (NanoZS, Malvern Instruments, UK). Transmission electron microscopy (TEM) (JEM-2100 Plus, JEOL, Japan) was conducted to investigate the morphology of the nanoparticles. The optical density of Nile red was measured using a microplate reader (PowerWave XS, Biotek, USA). To determine and then calculate the binding efficiency of the primers, the optical density of the primers in the supernatant was measured using a UV–vis spectrophotometer (NanoDrop 2000c, Thermo Fisher Scientific Inc., USA). The concentration of ETEC was determined by measuring the optical density using a UV‒vis spectrophotometer (Shimadzu, Japan). PCR amplification was conducted using a thermal cycler (Bio-Rad Laboratories, USA). Agarose gel electrophoresis was visualized using the Gel Doc™ EZ System (Bio-Rad Laboratories, USA). For MELGA, the optical density of oxidized TMB was measured at 650 nm using a microplate reader (Synergy HTX Multi-Mode Reader, BioTek, USA).

### Bacterial cultivation and DNA extraction

Bacterial isolates of ETEC (DMSC 25093) were recovered onto tryptic soy agar (TSA) and multiplied in tryptic soy broth (TSB). The concentration of bacteria was adjusted by measuring the optical density value of 600 nm to 0.5, which is equivalent to 10^8^ CFU/mL using a UV‒vis spectrophotometer. The exact concentration of ETEC at OD_600nm_ was 1.41 × 10^8^ CFU/mL. According to the instructions, bacterial DNA was extracted from the bacterial isolates using the Presto™ Mini gDNA Bacteria Kit. The bacterial DNA concentration was determined by measuring the optical density (260 nm) using a spectrophotometer.

### Synthesis of dye-loaded solvent-sensitive nanoparticles (SSNPs)

Aminated mesoporous silica nanoparticles (NH_2_-MSNs) (200 mg) were dispersed in Nile red (NR) solution (5% w/v in ethanol, 5 mL) and sonicated for 2 min. DI water (20 mL) was added and subsequently stirred using a magnetic stirrer for 24 h, allowing NR molecules to diffuse into the NH_2_-MSNs. The mixture was centrifuged to separate NR-doped NH_2_-MSNs (NH_2_-MSNs-NR) and excess dye and washed three times with DI water. The NH_2_-MSNs-NR were dried at 60 °C for 24 h. The surface of the NH_2_-MSNs-NR was then modified by grafting with PMAMVE. In brief, a freshly prepared PMAMVE solution (5 mg/mL in DMSO, 2 mL) was added to an NH_2_-MSNs-NR suspension (1% w/v, 18 mL). The mixture was stirred at 400 rpm and 37 °C for 3 h before centrifugation and washing with DI water. The PMAMVE-functionalized NH_2_-MSNs-NR (NH_2_-MSNs-NR-PMAMVE) or SSNPs were stored as a colloidal suspension in DI water (1% w/v) until use.

### Nanoparticle characterization

The characteristics of the prepared SSNPs, including hydrodynamic size, polydispersity index (PDI), and zeta potential, were determined by Nanosizer. The morphology of the nanoparticles was analysed using TEM. Furthermore, the encapsulation efficiency of NR was determined by measuring the optical density of the supernatant after the synthesis process at 400 nm. Then, the values were calculated according to Eq. (1) and the standard curve.

1 $${\text{Encapsulation}}\,{\text{ efficiency }}\left( \% \right) \, = \frac{{\left( {\left[ {{\text{dye}}} \right]{\text{total }}{-}{ }\left[ {{\text{dye}}} \right]{\text{supernatant}}} \right)}}{{\left[ {{\text{dye}}} \right]{\text{total}}}} \times 100$$

In addition, the SSNPs were tested for solvent response with various organic solvents. The SSNPs (1% w/v in DEPc-treated water, 500 µL) were mixed with organic solvents, including absolute ethanol, absolute methanol, acetone, ethyl acetate, DMSO (500 µL), and DI water (1 mL). The mixture was sonicated, mixed thoroughly for 0, 5, 10, 15, 20, 25, and 30 min, and subsequently spun down to separate the supernatant. The optical density of the supernatant of each fraction was analysed using a UV‒vis spectrophotometer in scanning mode. The amount of NR released was determined based on the calibration curve.

## Preparation of LT DNA primers immobilized onto the nanoparticles

### Immobilization of LT-F onto MNPs

The specific LT forwards primer modified with an amine group at the 5′ end (5′-NH_2_-C_6_-GGC GAC AGA TTA TAC CGT-3′, NH_2_-LT-F) was immobilized onto carboxylated magnetic nanoparticles (COOH–MNPs) (1.45 wt%, hydrodynamic size 250 nm, zeta potential − 40 mV) through the carbodiimide method^20^. First, COOH-MNPs (1 mg) were washed twice with MES buffer (25 mM, pH 6.0, 500 µL). After that, NH_2_-LT-F (5 nmol in MES buffer) was added to the carboxylated MNPs and incubated at 900 rpm and room temperature for 30 min. Freshly prepared EDC solution (10 mg/mL, 10 µL) was subsequently added to the solution, and the mixture was then continuously mixed at 900 rpm overnight at room temperature. The residual concentration of unbound primers was measured from the supernatant after centrifugation using a UV–vis spectrophotometer. The binding efficiency and amount of immobilized NH_2_-LT-F on the carboxylated MNPs were calculated according to Eqs. (2) and (3), where C_i_ and C_f_ are the initial and final concentrations of oligonucleotide in solution (ng/µL), respectively, Q is the amount of immobilized NH_2_-LT-F on the carboxylated MNPs (nmol of oligonucleotide/mg of MNPs), M is the molecular weight of NH_2_-LT-F (5702.8 g/mol), m is the mass of the MNPs (mg), and V is the volume of solution (µL)^19^.

2 $${\text{Binding efficiency }}\left( \% \right) = \left( {\frac{{{\text{Ci}} - {\text{Cf}}}}{{{\text{Ci}}}}} \right)100$$

3 $${\text{Q }} = \left( {\frac{{{\text{Ci}} - {\text{Cf}}}}{{{\text{Mm}}}}} \right){\text{v}}$$

The LT–F–MNPs were then mixed with Tris buffer (50 mM tris(hydroxymethyl) aminomethane, pH 7.4, 500 µL) for 15 min at room temperature. Next, the nanoparticles were separated from the supernatant, resuspended in TE buffer (10 mM Tris-HCl, 1 mM EDTA, pH 8.0, 50 µL) to obtain the final concentration of stock primer (100 µM) and stored at 4 °C.

### Immobilization of LT-R onto SSNPs

Amino-modified specific LT reverse primer in MES buffer (pH 5.0) (NH_2_-LT-R, 5 nmol) (5′-NH_2_-C_6_-CGG TCT CTA TAT TCC CTG-3′) was added into the synthesized SSNPs (1 wt%) and then mixed at 900 rpm and 37 °C for 3 h. Next, the samples were centrifuged at 900 rpm for 15 min. The supernatant was then collected to evaluate the binding efficiency and amount of immobilized NH_2_-LT-R on the SSNPs using Eqs. (2) and (3) (where *M* of LT–R is 5596 g/mol). Afterwards, the primer-immobilized nanoparticles or LT-R-SSNPs were washed twice with Tris buffer at room temperature and 900 rpm for 15 min, resuspended in TE buffer (50 µL) to obtain the final concentration of stock primer (100 µM) and stored at 4 °C.

### Conventional PCR

Conventional PCR was performed for limit of detection evaluation. A pair of primers were used to amplify a 450 bp segment of the heat-labile enterotoxin encoding gene (*LT*) of ETEC; these primers were designed using Primer-BLAST software and synthesized as previously reported^8,19^. Each sample was prepared in a mixture (50 µL) using 1 × Accustart II PCR Supermix (25 µL) containing 1.5 mM MgCl_2_, 0.2 mM dNTPs, 1.25 U of Taq DNA polymerase, 10 µM of each LT-F primer (5′-GGC GAC AGA TTA TAC CGT-3′) and LT-R primer (5′-CGG TCT CTA TAT TCC CTG-3′) (0.5 µL) and 25 ng/µL of DNA template (5 µL). DEPc-treated water was used as the negative control. PCR amplification was conducted using a thermal cycler under optimal conditions. The process was started with a 2 min initial cycle at 94 °C and followed by 35 cycles of 94 °C for 50 s, 54 °C for 30 s and 72 °C for 30 s. A final extension step was performed at 72 °C for 5 min. The PCR products were then analysed for 450 bp segments by agarose gel electrophoresis at 100 V for 30 min and visualized using the Gel Doc™ EZ System after staining with GelRed.

### PCR inhibition testing

PCR inhibition of *LT* gene amplification for the synthesized nanoparticles was tested. Different amounts of SSNPs, i.e., 10, 15, 20, 25, 30 µg, and 0 µg (as a positive control), were added to each tube of the PCR mixtures, and a nonbacterial sample was used as a negative control. Afterwards, all samples underwent conventional PCR using LT-F primers (5′-GGC GAC AGA TTA TAC CGT-3′) and LT-R primers (5′-CGG TCT CTA TAT TCC CTG-3′) under the optimal conditions as described above and were then analysed using agarose gel electrophoresis.

### MELGA

The MELGA technique was performed following the protocol of a previous study^8^. Each reaction was conducted in a mixture (50 µL) including 1 × Accustart II PCR Supermix (25 µL) containing 1.5 mM MgCl_2_, 0.2 µM dNTPs, 1.25 U of Taq DNA polymerase, LT–F–MNPs (1 µL), 10 µM biotinylated LT-R primer (biotin-5′-CGG TCT CTA TAT TCC CTG-3′) (LT-R-biotin) (1 µL) and 25 ng/µL ETEC DNA template (5 µL). MELGA proceeded under identical conditions for conventional PCR. The PCR product-bound MNPs (biotin-amplicon-MNPs) were captured by an external magnetic field, subsequently blocked with BSA solution (1% w/v in DEPc-treated water) and then incubated with horseradish peroxidase conjugated streptavidin (SA-HRP) (0.1 µg/mL, 50 µL) in a dark chamber at room temperature for 60 min to allow biotin and streptavidin to interact. MELGA products (HRP-SA-biotin-amplicon-MNPs) were washed twice with DEPc-treated water and incubated with TMB substrate (3, 3′, 5, 5′-tetramethylbenzidine) in the dark at room temperature for 60 min to complete the oxidation of TMB. Finally, the absorbance of oxidized TMB was measured at 650 nm using a microplate reader and compared to a DNA-free sample as a negative control. The result was interpreted as the relative optical density (ROD) according to Eq. (4), where ODS and ODN are the sample and negative control, respectively.

4 $${\text{ROD}} = \frac{{{\text{ODS}}}}{{{\text{ODN}}}}$$

### Optimization of the SSNP-enhanced PCR assay

The SSNP-enhanced PCR assay was carried out based on the MELGA protocol using 10 µM LT-F-MNPs (5′-MNPs-NH_2_-*C*_*6*_-GGC GAC AGA TTA TAC CGT-3′) (1 µL) and various amounts of LT-R-SSNPs (SSNPs-5′-CGG TCT CTA TAT TCC CTG-3′) including 1.0, 1.5, 2.0, 2.5, 3.0, 4.0, and 5.0 µL (equivalent to 10, 15, 20, 25, 30, 40 and 50 µg of SSNPs, respectively). For the postamplification step, the product bound onto MNPs (SSNPs-amplicon-MNPs) was sorted out using a magnet and was subsequently washed with BSA solution (1% w/v in DEPc-treated water), and the supernatant was discarded. Afterwards, a suitable organic solvent (200 µL) was added to the sample and subsequently mixed at 900 rpm for 15 min. After the supernatant from the samples was collected, the optical density was measured using a microplate reader with comparison to the negative control (DNA-free sample). The optical density values were calculated into the relative value (ROD) according to Eq. (4)^7^. The number of LT-R-SSNPs that provided the highest ROD was the best for the SSNP-enhanced PCR assay and was used in further experiments.

### Comparison of the detection limit

The limit of detection of conventional PCR, MELGA, and SSNP-enhanced PCR assays was evaluated and compared in terms of sensitivity. First, the ETEC DNA sample (25 ng/µL) was serially diluted tenfold into 8 fractions, including 25 ng/µL, 2.5 ng/µL, 0.25 ng/µL, 25 pg/µL, 2.5 pg/µL, 0.25 pg/µL, 25 fg/µL, and 2.5 fg/µL. PCR, MELGA, and SSNP-enhanced PCR assays of all samples at different concentrations were performed using primers alongside DNA-free samples as the negative control. For interpretation, the result of PCR gel electrophoresis was analysed by considering the DNA concentration of the last band (450 bp) that could be observed as a limit of detection of ETEC by conventional PCR. For MELGA and SSNP-enhanced PCR assays, all RODs were analysed by SPSS version 1.4.1 software (Free Software Foundation) using repeated-measures ANOVA. Post hoc analysis was applied using Dunnett’s multiple comparisons to test the sensitivity and detection limit. The mean (SEM) standard error values are represented as the thin line above the bars. The results were statistically significant when *P* < 0.05 at the 95% confidence interval (CI).

## Results

### Nanoparticle characterization

**Figure 2:**
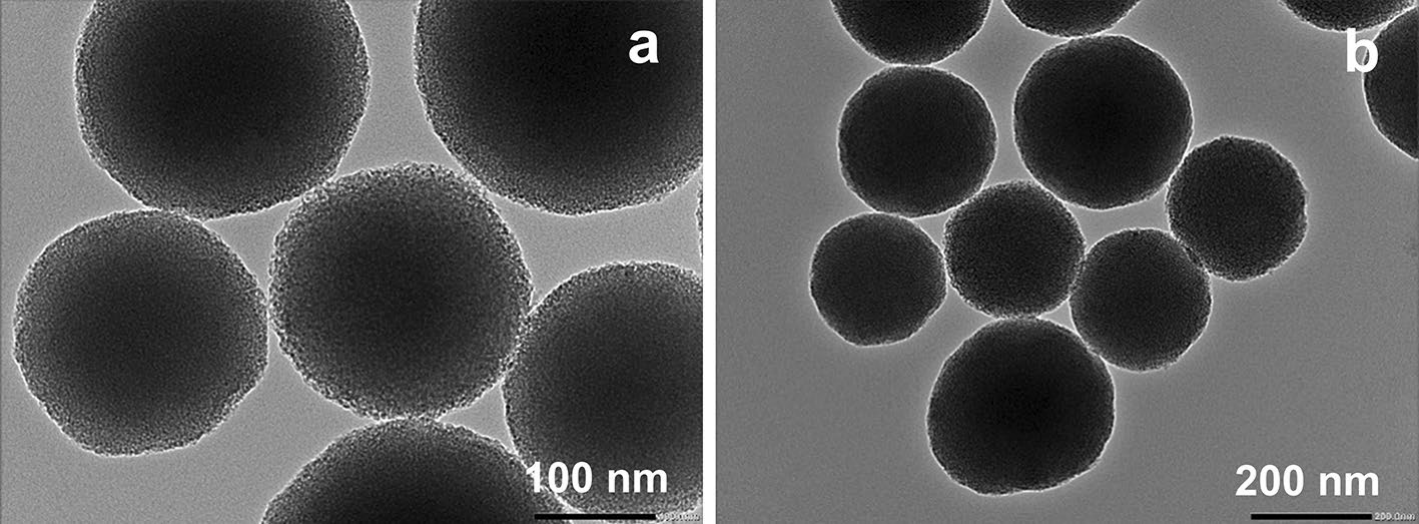
TEM micrographs of SSNPs (NH_2_-MSNs-NR-PMAMVE) at 100,000 × (**a**) and 50,000 × (**b**).

NH_2_-MSNs-NR-PMAMVE or SSNPs were used to measure the hydrodynamic size, size distribution, zeta potential, and encapsulation efficiency. According to the results in Table 1, the nanoparticles possessed a hydrodynamic size of 340.2 ± 3.4 nm with a narrow size distribution. The high absolute zeta potential value (> 30 mV) indicated that the nanoparticles exhibited good colloidal stability. Moreover, the morphology of the SSNPs was spherical (Fig. 2), as determined by TEM, and the particle size distribution was related to the results from dynamic light scattering. The particle size of the SSNPs in the dried state was 246.9 ± 28.6 nm, which was smaller than that determined using Nanosizer because PMAMVE shrinks in the dried state.

**Table 1:** The result of dynamic light scattering measurement of NH_2_-MSNs-NR-PMAMVE.

Sample	Hydrodynamic size (nm)	Zeta potential (mV)	PDI	Encapsulation efficiency
NH_2_-MSN-NR-PMAMVE	340.2 ± 3.4	−47.5 ± 1.0	0.09	85.68%

**Figure 3:**
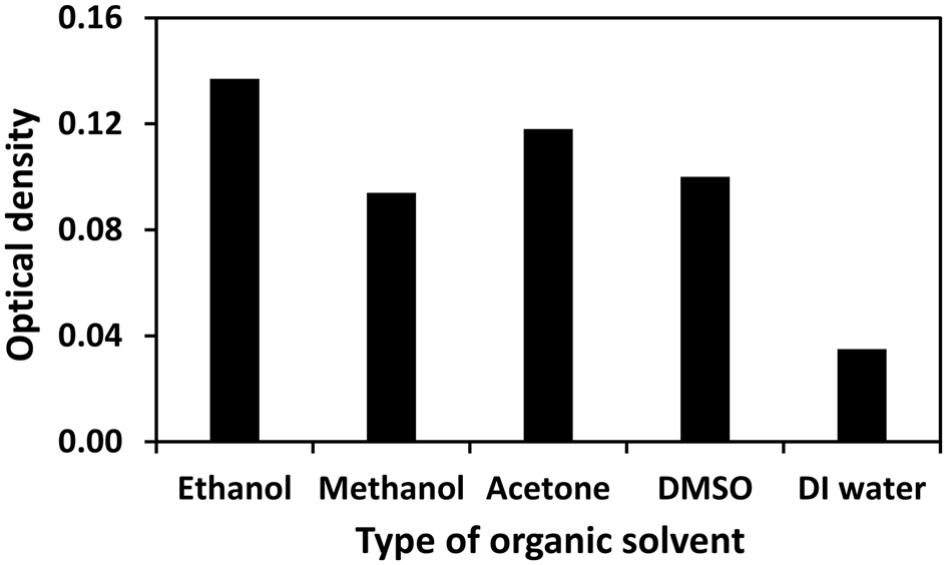
Comparison of optical density values indicating the efficiency of organic solvents in stimulating SSNPs. The optical density was measured at the maximum wavelength of NR in each solvent, which were 553 nm, 585 nm, 580 nm, 585 nm, and 400–600 nm for ethanol, methanol, acetone, DMSO, and DI water, respectively.

Stimulation testing was performed with the SSNPs using various types of organic solvents, including ethanol, methanol, acetone, ethyl acetate, and DMSO; this was performed to identify the fastest stimulating solvent through which SSNPs delivers a relatively intense optical density. The obtained results are displayed in Fig. 3.

**Figure 4:**
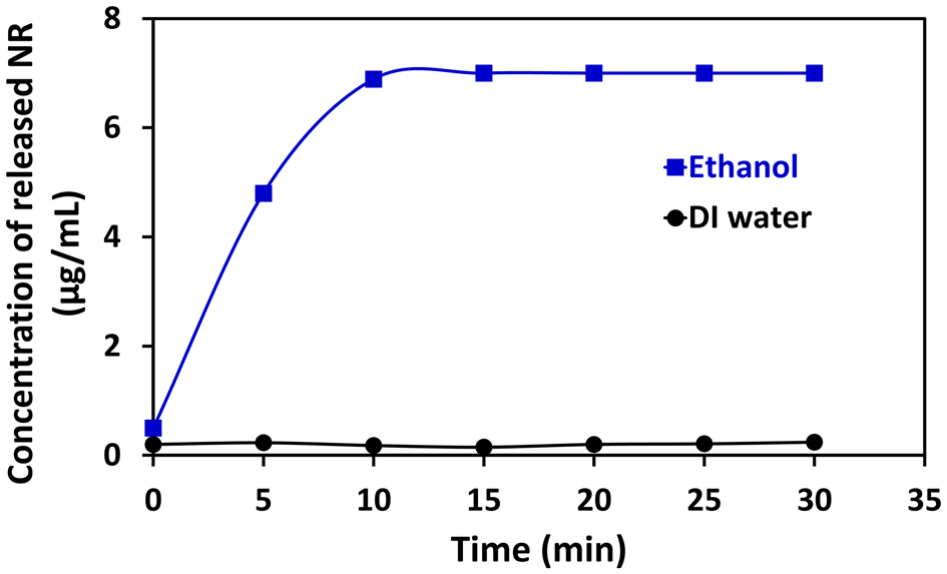
The concentration of released NR in ethanol (blue) compared with DI water (black) at different incubation times.

After the SSNPs were incubated in ethanol, the highest optical density of the supernatant was obtained, suggesting that ethanol was the most efficient stimulus of these nanoparticles among the candidates. Afterwards, the time of stimulation to the endpoint was evaluated. The SSNPs were mixed with ethanol and DI water and subsequently shaken continuously for different durations (0, 5, 10, 15, 20, 25, and 30 min). Then, the optical density measurements were obtained at 553 nm for the supernatant of each mixture, as presented in Fig. 4.

All values were calculated as the accumulated concentration of NR released into the mixture. The results showed that after 10 min, the amount of NR released reached a stable point. The results indicated that the optical density of NR release can be evaluated after adding ethanol to the SSNPs and mixing for 15 min.

### PCR inhibition testing of SSNPs

**Figure 5:**
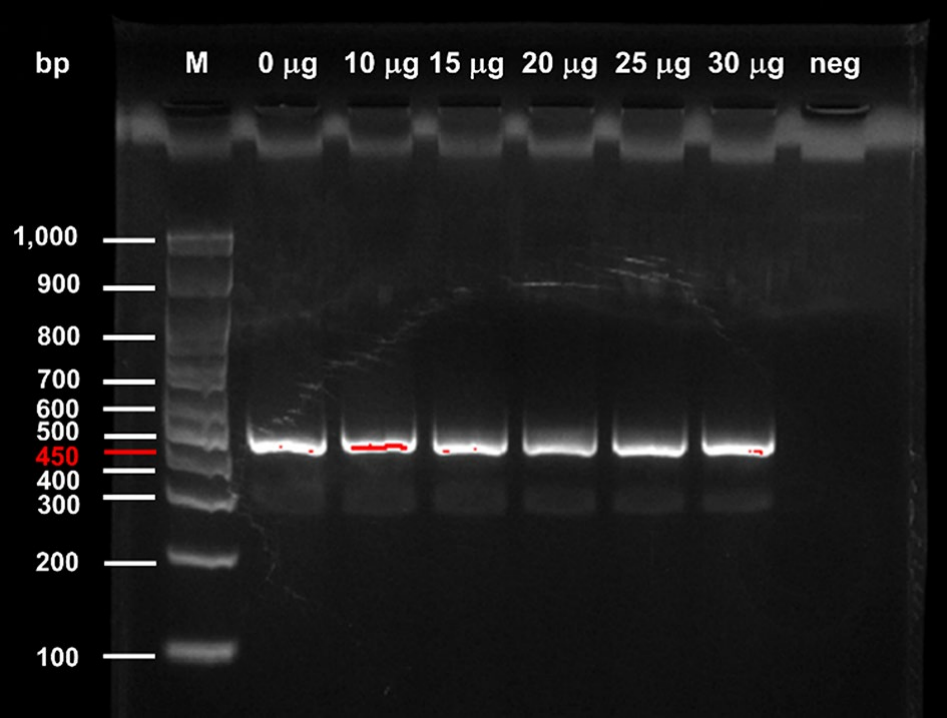
The result of gel electrophoresis for testing the inhibition of PCR by SSNPs at various amounts in *LT* gene detection (M = standard marker and neg = negative control). Original gel is presented in Supplementary Fig. 1.

The SSNPs are likely promising for this study due to their characteristics and response to the stimulus. Thus, the SSNPs in various amounts (0–30 µg/reaction) were further tested for compatibility with PCR while detecting the ETEC heat-labile enterotoxin gene. The results are displayed in Fig. 5.

The results showed that PCR inhibition was not detected in the presence of 10–30 µg of the SSNPs. This indicated that the SSNPs exhibited good compatibility with PCR.

### Immobilization of LT primers onto nanoparticles

By using EDC as a coupling agent in the carbodiimide method, NH_2_-LT-F was successfully bonded onto the carboxylated MNPs through covalent bonds. Likewise, NH_2_-LT-R was covalently bound to the SSNPs (NH_2_-MSNs-NR-PMAMVE) through interaction between the PMAMVE molecule on the surface of SSNPs as a coupling agent itself and amino groups in NH_2_-LT-R. The primers exhibited a high binding efficiency to each nanoparticle, as shown in Table 2, indicating that most LT-F and LT-R were effectively immobilized on the carboxylated MNPs and SSNPs, respectively. The oligonucleotide-immobilized nanoparticles could therefore be used in PCR-based assays.

**Table 2:** Result of immobilization of LT-F onto the carboxylated MNPs and LT-R onto the SSNPs.

Primer-particle	Initial concentration (ng/µL)	Residual concentration (ng/µL)	Number of immobilized primers on particles (nmol of primer/mg of NPs)	Binding efficiency (%)
LT-F-MNPs	285.14	49.5	5.8	82.64
LT-R-SSNPs	186.53	4.1	4.9	97.80

### Optimization of SSNPs-enhanced PCR assay

**Figure 6:**
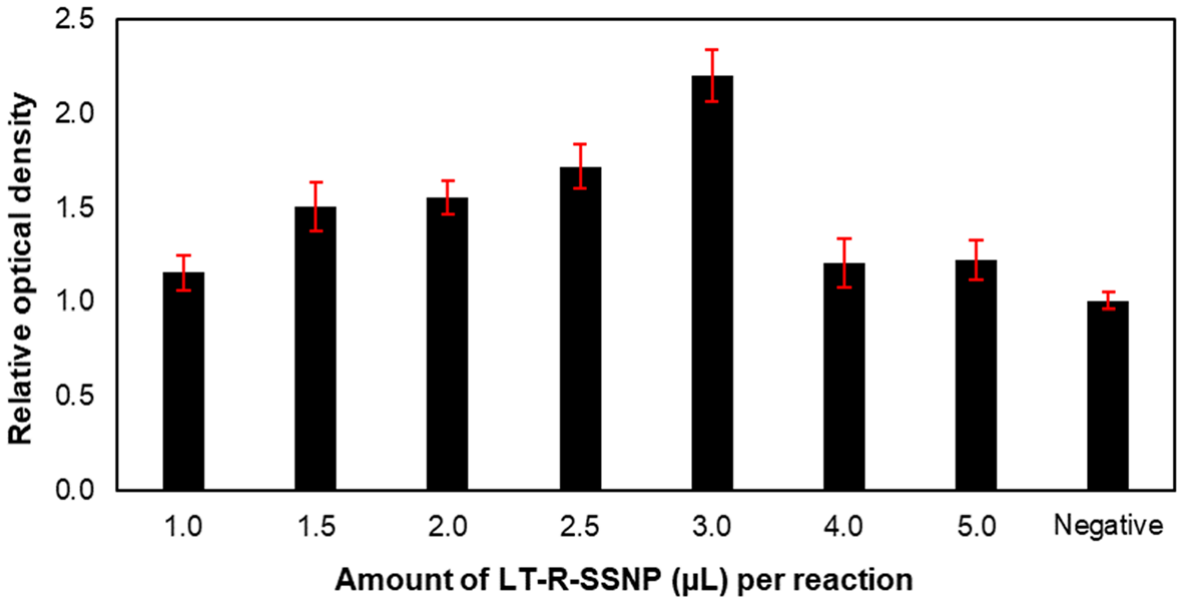
The result of ROD performed using different amounts of LT-R-SSNPs in the SSNP-enhanced PCR assay (Negative = DNA-free sample).

The optimal amount of LT-R-SSNPs was evaluated before performing this technique in the next experiments. The volume of LT-R-SSNPs added to the reaction was 1, 1.5, 2, 2.5, 3, 4, and 5 µL (corresponding to 10, 15, 20, 25, 30, 40 and 50 µg of SSNPs, respectively). After PCR, the addition of organic solvent, and optical density measurement, all values were calculated into ROD. The results indicated that with LT-R–SSNPs volumes of 1 to 3 µL, the ROD and concentration of LT-R-SSNPs were directly proportional. On the other hand, at LT-R-SSNPs volumes of 4 and 5 µL, the results showed no significant difference in ROD values compared to that of the negative control (DNA-free sample), as shown in Fig. 6. According to the results, 3 µL or 30 µg of LT-R-SSNPs was considered the best amount for the SSNPs-enhanced PCR assay for the next experiments.

### Comparison of the limit of detection

**Figure 7:**
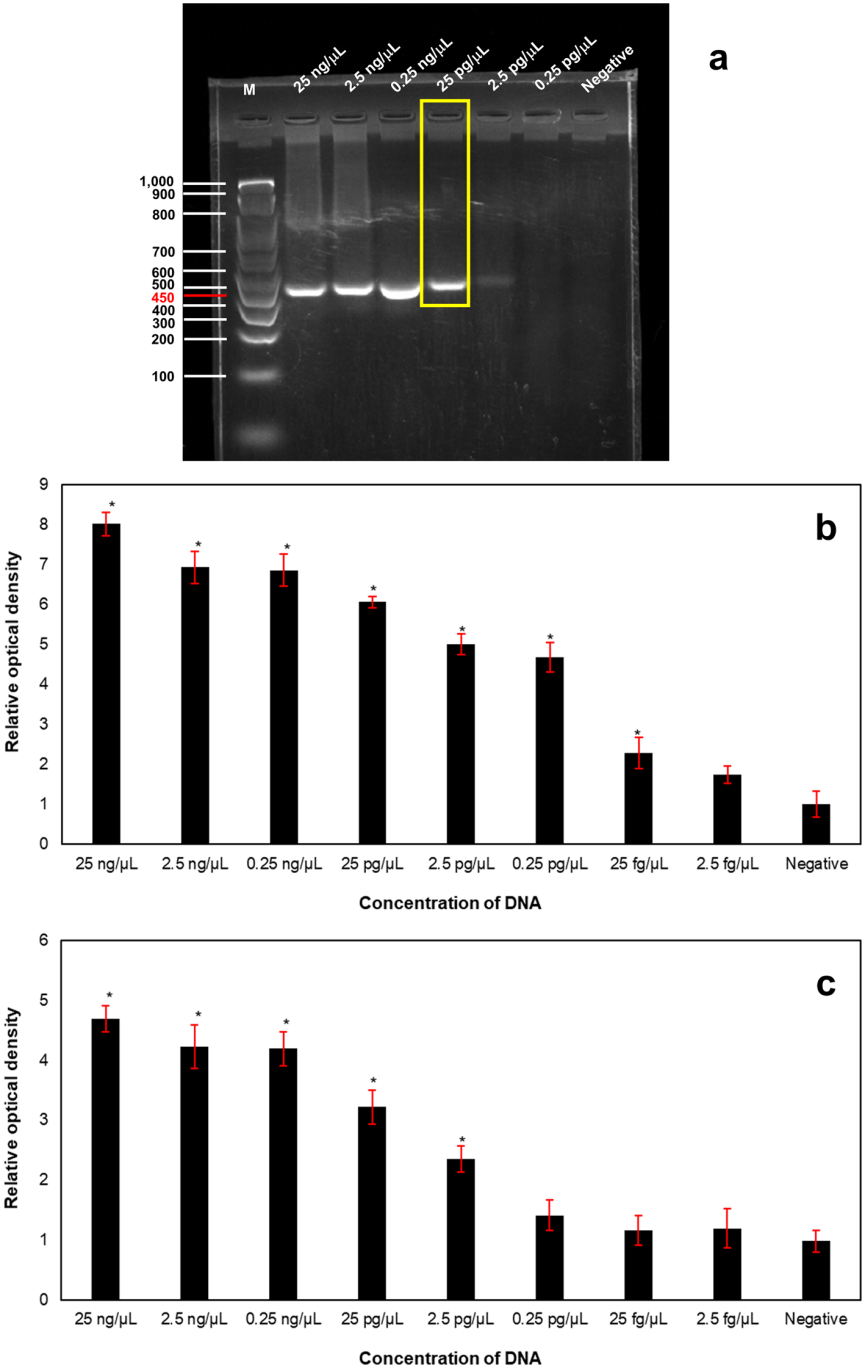
Evaluation of the detection limit for detecting the *LT* gene using conventional PCR (**a**), MELGA (**b**) and SSNP-enhanced PCR assays (**c**). Original gel of conventional PCR is presented in Supplementary Fig. 2.

Extracted DNA samples of ETEC were used in PCR-based assays at different concentrations (25 ng/µL, 2.5 ng/µL, 0.25 ng/µL, 25 pg/µL, 2.5 pg/µL, 0.25 pg/µL, 25 fg/µL, and 2.5 fg/µL); then, the detection limit of the samples was analysed and compared to each other. The detection limit evaluation for conventional PCR in the detection of the *LT* gene, which is defined as the final concentration of the DNA sample that provided a visible specific band of the target gene (450 bp), was 25 pg/µL (2.5 × 10^–11^ g/µL) (Fig. 7a). However, the detection limits of the MELGA and SSNP-enhanced PCR assays were 25 fg/µL (2.5 × 10^–14^ g/µL) (Fig. 7b) and 2.5 pg/µL (2.5 × 10^–12^ g/µL) (Fig. 7c), respectively.

## Discussion

Solvent-sensitive nanoparticles (SSNPs) composed of mesoporous silica nanoparticles (MSNs), Nile red (NR), and PMAMVE were synthesized as a structural support, signalling dye, and gatekeeper, respectively. PMAMVE copolymer is a good surface modification polymer that was reported to drastically improve particle stability and monodispersity; thus, PMAMVE contributes to successful amplification of the target DNA and is effectively used as a cross-linking agent^20^. The synthesized SSNPs with a hydrodynamic size of 340.2 ± 3.4 nm were accepted as homogeneous, as the PDI was less than 0.3. The high magnitude of the zeta potential value (− 47.5 ± 1.0 mV) of the nanoparticles suggested that the repulsive force was sufficient to maintain the physical colloidal stability upon storage. According to the results of the release profile, the optical density of the SSNPs stimulated in ethanol was highest. It was suggested that compared to other solvents used in the test, ethanol is the most effective stimulator with less than 15 min of stimulating time. A 15 min mixing period was the optimal time for inducing SSNPs to release the dye. When testing the inhibition of PCR by SSNPs, the sign of PCR inhibition was completely absent at 10 to 30 µg per reaction. Accordingly, the SSNPs were anticipated to be promising signalling support for postamplification analysis of a novel PCR-based assay.

The newly developed assay, the SSNPs-enhanced PCR assay, was first optimized for an optimal amount of LT-R-SSNPs in PCR. The amount of LT-R-SSNPs ranged from 1.0, 1.5, 2.0, 2.5, 3.0, 4.0, and 5.0 µL (equivalent to 10, 15, 20, 25, 30, 40 and 50 µg of SSNPs). After amplification, all samples were subsequently treated with ethanol and mixed for 15 min before the optical density of the supernatant was measured. The result suggested that 30 µg of LT-R-SSNPs provided the highest positive result for the optical density, whereas 40 and 50 µg showed no significant positive result because the PCR was inhibited from an excessive amount of particles^20–22^.

The detection limit of the three assays was evaluated for comparison. According to the results, the detection limit of MELGA appeared to be the lowest (10^–14^ g/µL), followed by the SSNP-enhanced PCR assay and conventional PCR (10^–12^ and 10^–11^ g/µL, respectively). Compared to conventional PCR, the SSNP-enhanced PCR assay was found to be 10 times more sensitive, while MELGA was 100 times more sensitive than the SSNP-enhanced PCR assay. This result agreed with the positive results observed with the naked eye, as the colour changes in positive samples during the SSNP-enhanced PCR assay could hardly be differentiated from negative samples. Despite the slightly lower sensitivity, the SSNP-enhanced PCR assay could provide a remarkably faster turnaround time (approximately 20 min after amplification) than that of MELGA or conventional PCR, as summarized in Table 3. Moreover, the developed assay is easier to perform in practise, and before adding a stimulus to develop a colorimetric signal, only a single washing step is needed. Hence, the assay can prevent a loss in target amplicons, leading to a decrease in false-negative results. Moreover, regarding aerosol contamination during processing, this assay is safer for users since the final product is DNA, which is inactivated. Nevertheless, sensitivity and specificity evaluations must be performed in clinical samples to assess the true sensitivity, repeatability, and reproducibility values. Since NR dye can produce a fluorescent signal, the amplicons can be detected through fluorescent spectroscopy to enhance the sensitivity of this approach. Moreover, the simplicity and time of the assay possibly can be improved for use in the field by combining the DNA extraction and amplification steps.

**Table 3:** Comparison performance of three PCR-based assays in the detection of the *LT* gene.

Assays	Post-amplification analysis			
	Approximate time (1–10 samples) (min)	Level of observability with naked eyes (weak–strong)	Limit of detection (g/µL)	Fluorescent measurement
SSNPs-enhanced PCR assay	20–30	Weak	10^–12^	YES
MELGA	100–120	Strong	10^–14^	NO
Conventional PCR	60	Strong	10^–11^	NO

## Conclusion

Solvent-responsive nanoparticles (SSNPs) were synthesized, and these nanoparticles were composed of mesoporous silica nanoparticles (NH_2_-MSNs) loaded with Nile red (NR) and were coated with a solvent-sensitive shell poly(maleic anhydride-*alt*-methyl vinyl ether) (PMAMVE) as a gatekeeper (NH_2_-MSN-NR-PMAMVE). The nanoparticles were monodispersed and colloidally stable in aqueous medium. After adding the nanoparticles into PCR, the amount of the SSNPs (ranging from 10 to 30 µg/reaction) did not inhibit the amplification reaction, as a band at 450 bp corresponding to the molecular mass of the PCR product was clearly observed. The PMAMVE acted as a good gatekeeper to control the fast release of NR from the nanoparticles within 10 min; in addition, the carboxyl groups in the PMAMVE structure facilitated the covalent coupling of amino-modified specific LT reverse primer (NH_2_-LT-R) with high coupling efficiency (~ 97.8%). Using various concentrations of target ETEC DNA, the detection limit of the proposed SSNP-enhanced PCR assay (10^–12^ g/µL) obtained was better than that of conventional PCR (10^–11^ g/µL) but poorer than that of MELGA (10^–14^ g/µL). Even though the limit of detection was higher for detecting ETEC than MELGA, the new assay possesses significantly faster postamplification analysis when compared to that of either conventional PCR or MELGA. The proposed assay reduced turnaround time by eliminating several washing, incubation and biotin-streptavidin interaction steps, resulting in a shorter turnaround time, less expensive reagents, and a simpler procedure in practice. Consequently, the SSNP-enhanced PCR assay can be a useful and promising PCR-based assay for detecting any target gene in the clinic.

## Supplementary Information

Supplementary Information.

## Data Availability

The datasets generated during the current study are available from the corresponding author on reasonable request.

## References

[1] Tang C (2020). Application of magnetic nanoparticles in nucleic acid detection. J. Nanobiotechnology..

[2] Bai X (2022). Rapid and accurate detection for *Listeria monocytogenes* in milk using ampicillin-mediated magnetic separation coupled with quantitative real-time PCR. Microchem. J..

[3] Chen Y (2020). Magnetic particles for integrated nucleic acid purification, amplification and detection without pipetting. TrAC - Trends Anal. Chem..

[4] Jangpatarapongsa, K. *et al.* Increased sensitivity of enterotoxigenic *Escherichia coli* detection in stool samples using oligonucleotide immobilized-magnetic nanoparticles. *Biotechnol. Rep. (Amst).* 32, e00677 (2021). 10.1016/j.btre.2021.e00677 PMC8487978 34631437

[5] Xu J (2021). A one step method for isolation of genomic DNA using multi-amino modified magnetic nanoparticles. RSC. Adv..

[6] Tangchaikeeree T, Polpanich D, Elaissari A, Jangpatarapongsa K (2017). Magnetic particles for in vitro molecular diagnosis: From sample preparation to integration into microsystems. Colloids Surf. B: Biointerfaces..

[7] Manthawornsiri Y (2016). Magnetic Nanoparticles PCR Enzyme-Linked Gene Assay for quantitative detection of *BCR/ABL* fusion gene in Chronic Myelogenous Leukemia. J. Clin. Lab. Anal..

[8] Jangpatarapongsa K (2011). DNA detection of chronic myelogenous leukemia by magnetic nanoparticles. Analyst..

[9] Jansaento W, Jangpatarapongsa K, Polpanich D, Wonglumsom W (2016). Detection of Campylobacter DNA using magnetic nanoparticles coupled with PCR and a colorimetric end-point system. Food Sci. Biotechnol..

[10] Ehi-Eromosele CO, Ita BI, Iweala EEJ (2017). Silica coated LSMO magnetic nanoparticles for the pH-responsive delivery of 5-Fluorouracil anticancer drug. Colloids Surf. A: Physicochem. Eng. Asp..

[11] Oh NM, Oh KT, Lee ES (2014). Development of pH-responsive poly(γ-cyclodextrin) derivative nanoparticles. Colloids Surf. B: Biointerfaces..

[12] Hosangadi D, Smith PG, Kaslow DC, Giersing BK (2019). WHO consultation on ETEC and Shigella burden of disease, Geneva, 6–7th April 2017: Meeting report. Vaccine..

[13] Wierzba TF, Bourgis A (2017). Defining cases of severe pediatric diarrhea for an efficacy trial of an enterotoxigenic *Escherichia coli* (ETEC) vaccine: Report on an international workshop, Washington DC, March 2016. Vaccine..

[14] Shahbazi G (2021). Characteristics of diarrheagenic *Escherichia coli* pathotypes among children under the age of 10 years with acute diarrhea. Gene. Rep..

[15] Byrd W, Boedeker EC (2013). Attenuated *Escherichia coli* strains expressing the colonization factor antigen I (CFA/I) and a detoxified heat-labile enterotoxin (LThK63) enhance clearance of ETEC from the lungs of mice and protect mice from intestinal ETEC colonization and LT-induced fluid accumulation. Vet. Immunol. Immunopathol..

[16] Kotloff KL (2017). Global burden of diarrheal diseases among children in developing countries: Incidence, etiology, and insights from new molecular diagnostic techniques. Vaccine..

[17] Dunbar, S. A., Zhang, H. & Tang, Y.-W. J. C. i. l. m. Advanced techniques for detection and identification of microbial agents of gastroenteritis. *Clin. Lab. Med.* 33, 527–552 (2013). 10.1016/j.cll.2013.03.003 23931837

[18] Gomes TAT (2016). Diarrheagenic *Escherichia coli*. Braz. J. Microbiol..

[19] Thiramanas R, Jangpatarapongsa K, Tangboriboonrat P, Polpanich D (2013). Detection of *Vibrio cholerae* using the intrinsic catalytic activity of a magnetic polymeric nanoparticle. Anal. Chem..

[20] Tangchaikeeree T (2017). Combination of PCR and dual nanoparticles for detection of *Plasmodium falciparum*. Colloids Surf. B: Biointerfaces..

[21] Bai Y (2019). A method based on amino-modified magnetic nanoparticles to extract DNA for PCR-based analysis. Colloids Surf. B: Biointerfaces..

[22] Bai Y (2015). Nanoparticles Affect PCR Primarily via surface interactions with PCR components: Using amino-modified silica-coated magnetic nanoparticles as a main model. ACS Appl. Mater. Interfaces..

