# Supplementary material for: Solvent-sensitive nanoparticle-enhanced PCR assay for the detection of enterotoxigenic Escherichia coli
Source: Sci Rep. 2022 Nov 30;12:20677. doi: 10.1038/s41598-022-25088-3 (PMC9712428; doi:10.1038/s41598-022-25088-3)
Supplement: Supplementary file 1 — Supplementary Information. [file 41598_2022_25088_MOESM1_ESM.docx]

**Supplementary Information**

Solvent-Sensitive Nanoparticle-Enhanced PCR Assay for the Detection of Enterotoxigenic *Escherichia coli*

**Patcharapong Teawprasong^1^, Yodsathorn Wongngam^2^, Tienrat Tangchaikeeree^1^,** **[Abdelhamid Elaissari](https://www.sciencedirect.com/science/article/pii/S0731708521002892?dgcid=coauthor#!)^3^, Pramuan Tangboriboonrat^4^, Duangporn Polpanich^2*^, Kulachart Jangpatarapongsa^1**^**

^1^ Center for Research and Innovation, Faculty of Medical Technology, Mahidol University,
Nakhon Pathom, 73170, Thailand. ^2^ National Nanotechnology Center, National Science and Technology Development Agency (NSTDA), Thailand Science Park, Pathum Thani, 12120, Thailand. ^3^ Univ Lyon, University Claude Bernard Lyon-1, CNRS, ISA-UMR 5280, 69622 Villeurbanne, FRANCE. ^4^ Department of Chemistry, Faculty of Science, Mahidol University, Rama 6 Road, Phyathai, Bangkok 10400, Thailand. Corresponding author: **Associate Professor Kulachart Jangpatarapongsa, Ph.D. E-mail: kulachart.jan@mahidol.edu. *Duangporn Polpanich, Ph.D. E-mail: duangporn@nanotec.or.th


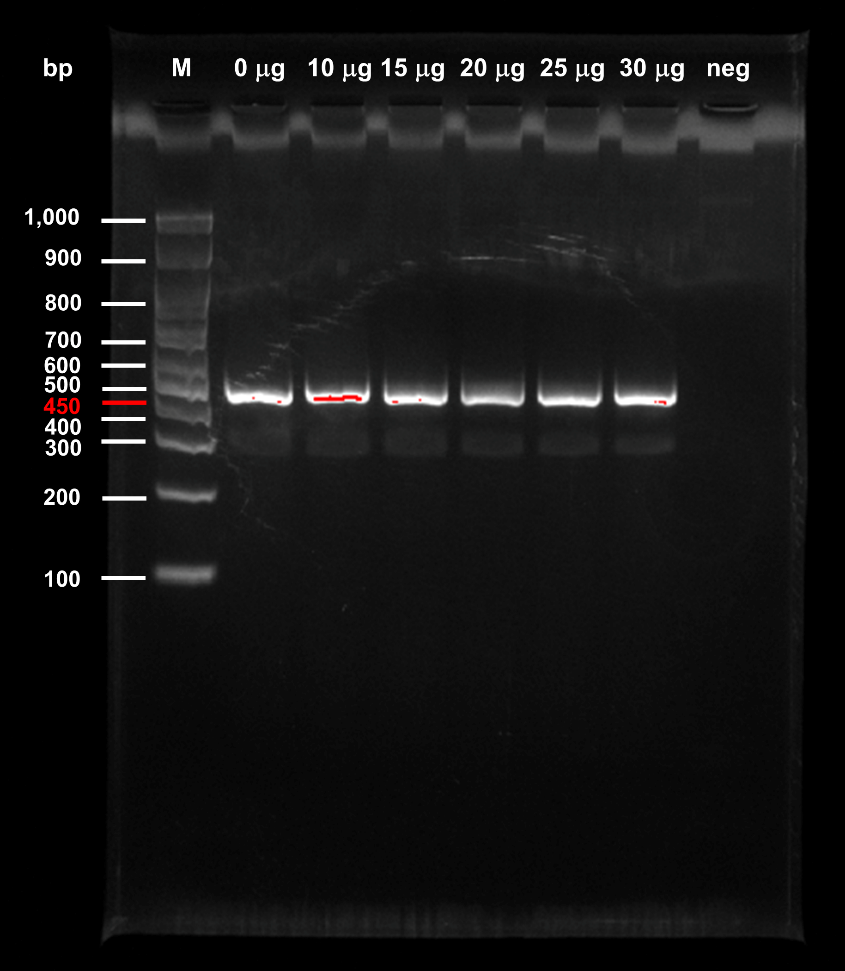


**Supplementary Figure 1.** The full-length membrane of gel electrophoresis for testing the inhibition of PCR by SSNPs at various amounts in *LT* gene detection (M = standard marker and neg = negative control).


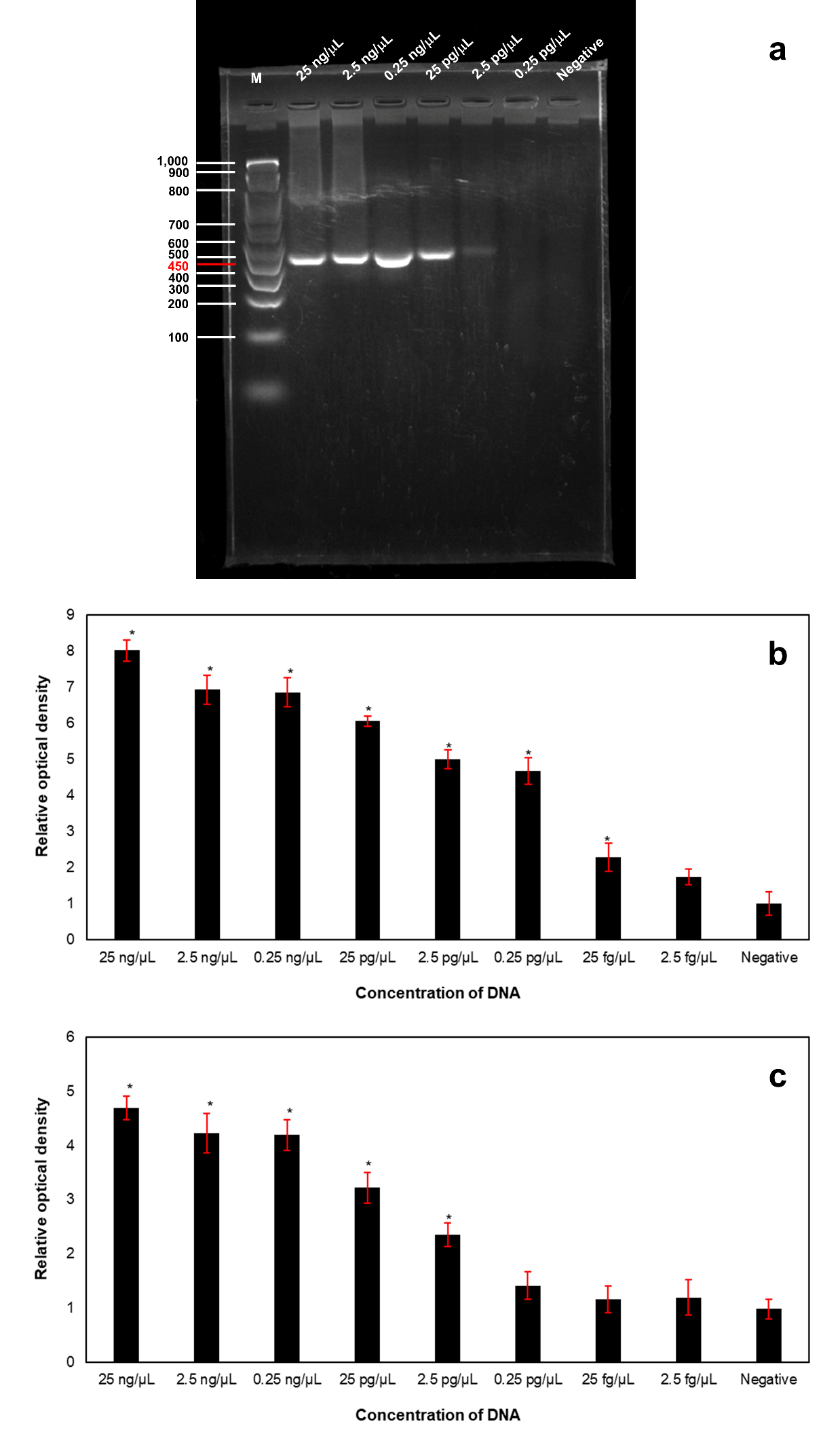


**Supplementary Figure 2.** The full-length membrane of gel electrophoresis for evaluation of the detection limit for detecting the *LT* gene using conventional PCR (M = standard marker and Negative = DNA-free sample).
